# Comment on “A Systematic Literature Review of Three Modalities in Technologically Assisted TKA”

**DOI:** 10.1155/2016/6539878

**Published:** 2016-04-28

**Authors:** Raju Vaishya, Nishint Gupta, Vipul Vijay, Amit Kumar Agrawal

**Affiliations:** Department of Orthopaedics, Indraprastha Apollo Hospital, Room No. 1210, Sarita Vihar, Mathura Road, New Delhi 110091, India

 We read with interest the recently published article in the journal discussing the systematic review of various new technologies for total knee arthroplasty. We have concerns about the published study [1] and would like to discuss it, along with our experience.

The present systematic literature review is based on the articles and data collected from single indexing source, that is, PubMed (MEDLINE) index, and other well-known indices like EMBASE, SCOPUS, and so forth have not been searched for the similar keywords. The relevance of this systematic review would have improved by the inclusion of other data bases.

The level of evidence of studies done for “kinetic sensor (KS)” is only either level III or level IV studies [2–5] which have been compared with level I or level II studies of the other two modalities, that is, Computer Assisted Orthopaedic Surgery (CAOS) and Patient Specific Instrumentation (PSI). Thus, this difference in the level of evidences of the studies between the two groups can lead to “selection bias” and hence the drawn inferences are not matchable and incorrect.

The authors have shown that it contributes upwards of $1,000 per procedure in vendor charges to the hospital (for the cost of fabrication of cutting blocks) and up to $1,000 in additional charges for imaging. In our setup, the CT imaging costs only $100, and the cost of manufacturing of customized cutting blocks comes to less than $400 [6, 7]. We believe that, with increasing use of PSI, the vendors shall be able to provide the manufacturing of cutting blocks locally, and this will help in reducing the cost further.

Although the authors have taken functional knee scores like KSS, WOMAC, and activity level scores for comparing the two modalities, any comparison of the postoperative mechanical axis was not included. Their conclusions have been drawn only from the postoperative functional knee scores like KSS, WOMAC, activity level scores, and so forth. It is well known that the primary objective of all these three technologically assisted TKA is to achieve a better and more accurate mechanical alignment postoperatively. Hence, not addressing this primary issue compromises the quality of this publication significantly.

Based on the above reasoning, we suggest that more level I and II studies are required to prove the efficacy of the KS for its use in TKA.
